# Promotion and tenure for medical physicists should be based on article specific measures and not on journal impact factor

**DOI:** 10.1002/acm2.14537

**Published:** 2024-10-10

**Authors:** Samantha Hedrick, Jinzhong Yang, Yi Rong

**Affiliations:** ^1^ Department of Radiation Oncology Thompson Proton Center Knoxville Tennessee USA; ^2^ Department of Radiation Physics the University of Texas MD Anderson Cancer Center Houston Texas USA; ^3^ Department of Radiation Oncology Mayo Clinic Arizona Phoenix Arizona USA

**Keywords:** academic promotion, journal impact factor, medical physics profession

## 1 INTRODUCTION

In the evolving progress of academic medicine, the metrics by which we measure success are both crucial and contentious. Among these, the Journal Impact Factor (JIF) has long been a dominant metric, often serving as a shorthand for the quality and significance of research. For many institutions, JIF plays a pivotal role in decisions regarding promotion, tenure, and funding, positioning it as a key indicator of academic achievement. However, as we delve deeper into the complexities of scholarly impact, questions arise: Is the JIF truly a fair measure of quality of an individual author or value of their article? Or should we, instead, focus on article‐specific metrics that more accurately reflect the true impact of the work? This month's debate seeks to explore these questions from both perspectives. We have Dr. Samantha Hedrick, who argues in favor of article‐specific measures as a more accurate reflection of scholarly contribution, while Dr. Jinzhong Yang defends the established role of the JIF as a useful, if imperfect, tool in academic evaluation.

Samantha Hedrick, PhD, DABR received her B.S. in Nuclear Engineering from the University of Missouri‐Rolla and received her M.S. and PhD in Nuclear Engineering from the University of Missouri. She then completed a two‐year CAMPEP accredited residency at Washington University in St. Louis. She is currently the Director of Medical Physics at the Thompson Proton Center, specializing in pencil beam scanning proton therapy treatment planning, scripting, and safety improvements.

Jinzhong Yang, PhD is an Assistant Professor in the Department of Radiation Physics at the University of Texas MD Anderson Cancer Center. He is the lead physicist of the MR‐Linac program at MD Anderson. He earned his PhD in Electrical Engineering from Lehigh University in 2006 and received a postdoctoral training at University of Pennsylvania. His research focuses on artificial intelligence in medical image computing for radiation oncology applications, MR‐guided online adaptive radiotherapy, and quantitative imaging biomarkers for treatment outcome prediction. He has published over 130 peer‐reviewed journal articles, nine book chapters, and edited a book.

### 1.1 Opening statement

#### 1.1.1 For the proposition: Samantha Hedrick, PhD

In the scientific community, publications can have an impact on promotion, tenure, grant funding, and more, and not all publications are considered equal. Typically, the Journal Impact Factor (JIF) is used to determine the quality of a publication. A publication in a journal with a high JIF is considered to be better than publishing in a journal with a lower JIF. SciJournal.Org reports that, for health professions, the average JIF is 2.02, with the top 20% averaging 2.72.^1^ The JIF was originally created as a tool to help librarians know which journals to purchase, rather than to indicate quality, and is based on the citation rate of articles over 2 years.^2^ This metric can be skewed by journals and authors that self‐reference and journals that publish many review articles, and the data used to calculate the JIF is not openly available to the public for review. The JIF does not account for the impact of individual articles or authors. A single publication that was highly discussed in a scientific community or influenced policy change could be “ignored” if only using the JIF to determine quality. Similarly, the h‐index has been used as a surrogate for the quality of a scientist. The h‐index counts the number of publications for which an author has been cited by other authors at least that same number of times.^3^ Like the JIF, this metric can be easily manipulated with self‐citations and review articles and is limited by the total number of publications for an author.

Considering that the JIF and h‐index are based on citations and number of publications, there can start to be a “publish or perish” mentality. As quantity starts to be valued over quality, predatory journals start to look more attractive. These journals are “pay to publish” with limited‐to‐zero peer review, low standards for research practices, and can be an easy, if expensive, way to bulk up a CV. Promotion and grant funding should be based on the quality of a scientist, not how much they can afford. That brings up the question of how to define quality. The San Francisco Declaration on Research Assessment (DORA), developed in 2012 during an annual meeting of The American Society for Cell Biology, proposes that the quality of a scientist should be defined by more than publishing in a competitive journal.^4^ DORA recommends to “consider a broad range of impact measures including qualitative indicators of research impact, such as influence on policy and practice” and to “encourage a shift toward assessment based on the scientific content of an article rather than publication metrics of the journal in which it was published”. Other scholarly products, besides only journal articles, should be considered, such as datasets, protocols, software, preprints, policies, and training.

DORA defines two dimensions that can be used to illustrate the impact of a physicist and their work; the scale of influence and new audiences. When considering a physicist for promotion or tenure, their facility could consider their scale of influence. Increasing scale of influence is advancing from journal articles, to teaching and mentoring, to leadership roles in societies or boards. When considering a physicist's publications, instead of using h‐index or JIF, the facility could consider the ability of the physicist to reach new audiences outside of their discipline, considering if they published in an open access journal or a press book. Evaluating these components can be challenging, as they are qualitative rather than quantitative, but they should not be ignored.

All of this is not to say that we cannot still use quantitative measures. There are new metrics being developed, such as the Eigenfactor, to try to process citations data in a new way that might provide a better measure of journal quality.^5^ Considering our new world of social media and online presence, the Altmetric score uses “mentions” from news sources, social media, patents, policy documents, and other online sources to quantify the amount of attention an article receives.^6^ This is not a measure of quality, but it can be a way to quantify a physicist's ability to increase their scale of influence and reach audiences.

Eliminating the focus on JIF and developing new evaluation tools will incentivize the actions of a “good” physicist, one who performs ethical research, shares their work with open access, develops collaborative relationships, strives towards societal impact, and mentors the next generation.

#### 1.1.2 Against the proposition: Jinzhong Yang, PhD

JIF is an index reflecting the mean number of citations of articles published in the last two years for a journal. JIF is a journal‐level metric, calculated annually by Clarivate (previously known as Institute for Scientific Information [ISI]) for journals indexed by Calrivate's Web of Science.^7^ The JIF was first introduced by Eugene Garfield in 1960s, with the purpose to help university librarians to select journals to purchase, particularly for those small (in terms of total number of articles published) but influential journals.^2^, ^8^ Over the past several decades, JIF has become increasingly important regarded by different scientific academic communities. The JIF is commonly used to evaluate the relative importance of a journal in a field, with a belief that a higher JIF indicates more important for the journal.^9^ It is widely used by researchers to decide where to publish. Also, due to its straightforward definition and ease of understanding, JIF has become a key role in assessing individual researchers in their job application, promotion, and funding proposals, etc.^10^

Although the original definition of JIF does not mean to evaluate the journal quality, studies have found that JIF has correlations with journals’ quality and reputation. Saha et al.^11^ surveyed physicians specializing in internal medicine in the United States to rate the quality of nine general medical journals and found that the correlation between JIF and physicians' ratings of journal quality was strong. This study suggested that JIF may be a reasonable indicator of quality for general medical journals. In a recent study, researchers investigated the relationship between JIF and the thoroughness and helpfulness of peer reviews, and they found that peer review in journals with a higher JIF tends to be more thorough, particularly in addressing study methods while giving relatively less emphasis to presentation or suggesting solutions.^12^ This partially suggests that a higher JIF may indicate a better peer‐review quality. Another study found that paper acceptance rate and JIF were negatively correlated,^13^ showing that a paper sent to a journal with a higher JIF potentially experienced a stricter peer‐review process. On another recent study, researchers screened intervention reviews from the Cochrane Database of Systematic Reviews and looked for well‐appraised meta‐analyses.^14^ In 2459 results from 446 meta‐analyses, they found that results with a higher JIF were on average closer to truth than those with a lower JIF. This study demonstrates that a journal with a higher JIF likely publishes higher quality papers.

Yet still, there are numeral critiques regarding the JIF usage. The most common criticism is the use of JIF for application review, promotion, and tenure (RPT). Some researchers believed that the extent of JIF for the evaluation of the quality of a specific article or journal for the evaluation of individual and collective research achievements is inappropriate.^15^, ^16^ There are also concerns of manipulation of JIF to increase journal impacts by some professionals of the scientific publishing industry.^17^ Despite all these criticisms, JIF is still widely used for promotion and tenure. As described by Hoeffel,^18^ “Impact Factor is not a perfect tool to measure the quality of articles but there is nothing better and it has the advantage of already being in existence and is, therefore, a good technique for scientific evaluation.” In 2019, a group of researchers published their investigation of how often and in what ways the JIF was used in universities from the United States and Canada.^19^ In their findings, “40% of research‐intensive institutions and 18% of master's institutions mentioned the JIF, or closely related terms. Of the institutions that mentioned the JIF, 87% supported its use in at least one of their RPT documents, 13% expressed caution about its use, and none heavily criticized it or prohibited its use. Furthermore, 63% of institutions that mentioned the JIF associated the metric with quality, 40% with impact, importance, or significance, and 20% with prestige, reputation, or status.”

In summary, although JIF was not originally designed to evaluate the journal quality, studies have shown that JIF might be positively related to journal peer‐review quality, journal paper quality, and journal reputation and impacts in a specific field. Using JIF for promotion and tenure has been well established in many academic institutions, with the knowledge of its limitations in promotion and tenure evaluation. In addition, JIF information is straightforward and easily accessible. Therefore, it is safe to use JIF for promotion and tenure for medical physicists respecting published articles.

### 1.2 Rebuttal

#### 1.2.1 For the proposition: Samantha Hedrick, PhD

Dougherty et al quoted it well, saying that “Goodhart's Law states that a measure ceases to be a good measure when it becomes a target.”^20^ While there is data supporting the potential link between JIF and author or journal quality, the JIF is decreasingly effective as authors target a higher metric value. Smaldino et al found that incentivizing higher citation rates can lead to poorer scientific methods.^21^ This seems to indicate that use of the JIF in RPT could actually decrease the quality of promoted scholars. Additionally, use of JIF for RPT could perpetuate already existing barriers. Studies have shown that women as sole, first, and last‐authors receive fewer citations than male authors in the same position.^22^, ^23^ Other studies have shown that non‐white first‐authors receive fewer citations than white authors.^24^ Women and racial minorities are already underrepresented in scholars on a tenure‐track,^25^, ^26^ and using the JIF for RPT will likely not improve their representation.

An ideal candidate for promotion and tenure is a scholar who will produce high‐quality research and contribute to the advancement of their scientific community. Basing a candidate's projected value on the number of citations they have received is simple but limited. Moher et al suggested six principles to guide research evaluation that focus on how a scholar's research was performed, rather than what was produced.^27^ While these suggestions, and those of DORA, are more challenging to assess than JIF, they are more likely to foster better scientific practices and produce higher quality scholars.

#### 1.2.2 Against the proposition: Jinzhong Yang, PhD

There are two major criticisms from Dr. Hedrick in using JIF in application RPT. One criticism is that the JIF does not account for the impact of individual articles or authors. While I partially agree with this statement, a fair argument is that publishing a paper at a reputational journal with a high JIF likely implies a high quality of the author's research work. In my opening statement, it has been shown that JIF is correlated with journals’ quality and reputation, including peer‐review process.^11^, ^12^, ^13^, ^14^ Another criticism is that the “pay for publish” predatory journals may manipulate the JIF by using self‐citations and publishing review articles for potential higher citations, thus increasing the JIF. Therefore, Dr. Hedrick suggested using article‐level metrics, often referred to as “altmetrics”,^28^ as advocated by DORA, as a substitute for JIF in RPT. I agree with Dr. Hedrick that publications in these predatory journals often have a low quality even if the journals have a high JIF and using these journals’ JIF in RPT may not appropriately evaluate the quality of research by the candidates. Altmetrics may be a great addition to evaluating the impact of an article; however, I would like to make my opponent aware that altmetrics can not address the quality concerns of publications.

First, using altmetrics cannot avoid citation manipulation. It should be noted that to manipulate a paper's citation is much easier than to manipulate a journal's citation. In addition, altmetrics capture different impacts of a paper in addition to citations, such as paper views/downloads, social media discussions, reference manager bookmarks, etc. A high altmetrics does not necessarily mean a high quality of paper, and certainly RPT reviewers cannot rely on altmetrics, for example, the number of views or downloads.^29^, ^30^ Second, a predatory journal with citation manipulation is more easily identified than a single paper citation manipulation. A predatory journal often has strong characteristics, such as pay for publication, false or misleading information, and aggressive and indiscriminate solicitation.^31^ Papers published at predatory journals should be excluded from RPT, or should signal an alert of negative quality of the candidates. There is a concerted effort to create lists of predatory journals, such as Beall's list,^32^ Cabells predatory report,^33^ and early warning journal list published by Chinese Academy of Sciences.^34^ Third, altmetrics emphasizes the influence of an article. This metric encourages article sharing and open access. To increase the impact, authors may put most effort in advertising the paper, such as preprint or social media sharing, rather than focusing on improving paper quality. Over‐emphasizing altmetrics is more likely to produce “high” impact but useless papers. In addition, open access often means pay for publication. One characteristic of predatory journals is pay for publication. On the other word, altmetrics encourages open access, or pay for publication, essentially promoting publications in predatory journals. Finally, Dr. Hedrick also mentioned that datasets, protocols, software, preprints, policies, and training should be considered in RPT. However, these items are often published without any peer‐review to guarantee quality. Using these publications in RPT could be subjective and biased.

As I mentioned in my opening statement, JIF is not a perfect tool to measure the quality of articles but there is hardly anything better. Using JIF for promotion and tenure has been well established in many academic institutions, with the knowledge of its limitations in promotion and tenure evaluation. A major concern from my opponent is the predatory journals that have low paper quality but high JIF and can mislead RPT reviewers. The lists of predatory journals^32^, ^33^, ^34^ can serve for academic institutions to exclude them in RPT consideration or raise alert of the candidate's qualification.

## CONFLICT OF INTEREST STATEMENT

The authors declare no conflicts of interest.
